# Wholegrain triticale sourdough: Effects of triticale:Wheat flour ratio and hydration level on bread quality

**DOI:** 10.1002/fsn3.4050

**Published:** 2024-03-24

**Authors:** Valeria Messina, Jason Cano, Anthony Silvio, Angela L. Pattison, Thomas H. Roberts

**Affiliations:** ^1^ School of Life and Environmental Sciences University of Sydney Camperdown New South Wales Australia; ^2^ Brasserie Bread Banksmeadow New South Wales Australia; ^3^ Plant Breeding Institute University of Sydney Narrabri New South Wales Australia; ^4^ Sydney Institute of Agriculture University of Sydney Eveleigh New South Wales Australia; ^5^ Present address: Vannella Cheese Marrickville New South Wales Australia

**Keywords:** moisture, sourdough bread, texture, triticale, wholegrain flour

## Abstract

Triticale (×*Triticosecale*) is a hybrid between wheat (*Triticum* spp.) and rye (*Secale cereale*), producing higher grain yields than wheat in challenging environments. Triticale grain is also highly nutritious. Thus, the potential of triticale grain for an expanded range of food applications should be explored. Sourdough bread has unique functional and nutritional properties, but understanding the effects of partial substitution of triticale for wheat flour and varying levels of dough moisture content on sourdough quality requires further research. The aim of this study was to evaluate the wholegrain flour of contrasting triticale cultivars in comparison to that of a common wheat cultivar in commercial sourdough breadmaking. Two triticale cultivars (Goanna and Hawkeye, selected from a panel of Australian genotypes) and one wheat cultivar (Scout) were grown in a field trial in northern NSW, Australia. Differences in quantitative texture and color parameters of the dough and sourdough bread resulting from (1) substitution of commercial wholemeal wheat flour with different proportions of wholegrain triticale or wholegrain wheat (Scout) flour (0%, 60%, 70%, 80%, and 90%) and (2) varying the amount of water included in the dough preparation (70%, 80%, 90%, and 100 g water/100 g flour) were determined. Replacement of wholemeal wheat flour with 60% wholegrain Goanna flour (protein content 12.76%; c.f. 11.50% for Hawkeye, 12.40% for Scout), and addition of 100 g water/100 g flour in the dough preparation gave the highest quality sourdough bread based on specific volume, texture, and color parameters and had similar properties to the control made from wheat alone.

## Graphical abstract

Triticale grain is highly nutritious and should be tested in an expanded range of foods. In this study, the effect of partial substitution of wholemeal wheat flour with wholegrain triticale flour, as well as the effect of varying dough water content, on quality parameters of sourdough bread was tested. The replacement of wholemeal wheat flour with 60% wholegrain triticale cv. Goanna flour and the addition of 100 g water/100 g flour in the dough preparation gave the highest quality sourdough bread based on specific volume, texture, and color.

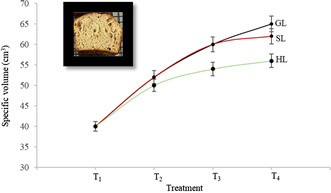

**Practical application**

Triticale is a hybrid cereal developed by breeders to combine the grain quality of wheat with the disease resistance and tolerance to harsh environments of rye. Triticale grain is highly nutritious, being rich in micronutrients and essential amino acids. For sourdough breadmaking, it is important to understand the effects on bread quality of varying the ratio between triticale and wheat flour, as well as the addition of different levels of water to the dough.

## 1 INTRODUCTION

Triticale was developed by crossing rye (the male parent) with wheat (the female parent) to achieve a combination of crop tolerance to harsh growing conditions (i.e., resistance to abiotic stress) and disease resistance from rye, and high yield, baking quality, and nutritional quality from wheat (McGoverin et al., 2011). Triticale grain is richer in vitamins, minerals, phosphorus, and essential amino acids, such as lysine, compared to grains of other cereals (Silva et al., 2020; Zhu, 2018). The valuable nutritional composition of triticale grain has made this hybrid cereal a replacement option for wheat (Galoburda et al., 2020; Goryanina, 2020).

The most common bread recipe consists of cereal flour, water, salt, and a leavening agent (Plessas, 2021; Yildrime & Arici, 2019), while the most common cereal flour used in breadmaking is wheat, which is rich in starch and has a higher protein content when compared to rice, maize, and rye (Järvan et al., 2017; Wang et al., 1999; Zörb et al., 2018). Moreover, the protein in wheat flour is mostly gluten, which is lacking in other cereals, and has the unique property of providing a viscoelastic dough critical for leavened bread. Substituting wheat flour with triticale flour is known to have an impact on the sensory and textural qualities of bread, including overall appearance, texture, volume, color, taste, and aroma (Fraś, Gołębiewska, Gołębiewski, Mańkowski, & Szecówka, 2016; Fraś, Gołębiewski, Gołębiewska, Mańkowski, Boros, et al., 2016). Replacement of a high proportion of wheat with triticale flour generally has a negative impact on sensory qualities, with bakery products becoming darker, stickier, and harder (Fraberger et al., 2020). Due to these potential effects, it is important to evaluate the optimum degree of wheat replacement with triticale to maintain bread quality and, therefore, consumer acceptance.

Sourdough fermentation, which began in ancient times and is still widely used in the baking industry (Chavan & Chavan, 2011), is based on the fermentation of dough by a combination of lactic acid bacteria (LAB) and yeast. Couch (2016) reported that the selection of different species of yeast and bacteria for sourdough breadmaking depends on the region of the world in which the bread is produced and the types of flour it contains. For example, *Lactobacillus sanfranciscensis* dominates the sourdough microflora of sweet leavened baked products and wheat breads produced in Italy (Alfonzo et al., 2016), and the same species has been found in traditional Greek wheat sourdough (Papadimitriou et al., 2019). *Lactobacillus pentosus* and *L*. *plantarum* dominate the sourdough breads produced in southern Italy that use durum wheat flour (Petrova & Petrov, 2020; Ventimiglia et al., 2015), while *L*. *brevis* is predominant in Turkish and Portuguese sourdough bread (Siepmann et al., 2018). *Lactococcus lactis* in sourdough bread fermentation has been reported to improve the sensory attributes of the bread. Zlateva et al. (2017) showed that applying a culture starter with *Lactococcus lactis* to sourdough wheat flour bread increased the biological value of the bread, extended shelf life, and improved sensory quality. Similar results were reported by Wu et al. (2022), who also used *Lactococcus lactis* as a culture starter in sourdough wheat bread and demonstrated a positive effect on the concentrations of aldehydes, ketones, and furans and improved sensory attributes.

Sourdough fermentation produces a bread of superior quality to standard leavened bread in terms of texture, flavor, nutritional attributes, and delaying microbiological spoilage. These improvements are due to specific proteins, including enzymes, as well as metabolites, including organic acids, exopolysaccharides, and even antimicrobial compounds, which are provided by the microbes in the dough (Demirkesen‐Bicak et al., 2021; Gänzle & Zheng, 2019; Gobbetti et al., 2019). The metabolites can also contribute to the relatively low glycemic index of sourdough bread via a lowering of starch digestibility (Arora et al., 2021; D'Alessandro & De Pergola, 2014; De Vuyst et al., 2016). Overall, using sourdough fermentation leads to a higher specific volume (i.e., loaf volume), a softer and more elastic structure, and a longer shelf life than for standard leavened bread (Ma et al., 2021). Indeed, the use of sourdough as a leavening agent for food fermentation is considered the gold standard (Gobbetti et al., 2019).

Bread quality is partly a reflection of the softness/firmness of the crumb, which is influenced by the complexes between starch and protein. When wheat gluten is compared to the rye storage proteins (secalins and prolamins), the latter are found to be unable to create a dough structure involving starch‐gluten complexes because of the lack of low‐molecular‐weight glutenin subunits; however, intermolecular disulfide bonds can still form among the rye storage proteins (Wang et al., 2017). Also, the presence of water‐extractable arabinoxylans in rye flour plays an important structural role via the binding of water and forming a viscous dough. The low pH of rye sourdough increases the extractability and swelling properties of the arabinoxylans (Wang et al., 2016).

One of the main functions of a sourdough culture in rye breadmaking is to inactivate α‐amylase (Mihhalevski et al., 2012). Under normal leavening conditions, rye has greater activity of α‐amylase than wheat; thus, inactivating this enzyme is particularly important in rye. The inactivation of α‐amylase via sourdough fermentation ensures that staling is slower than in normally leavened wheat breads (Deleu et al., 2020).

The aim of this research was to determine the effects on the texture and color properties of triticale sourdough bread made using (1) different ratios of wholegrain triticale flour to commercial wholemeal wheat flour and (2) different levels of water content in the dough (i.e., dough yield, defined as 100 parts of flour plus the amount of water (in parts) used for hydration). Our hypothesis was that sourdough bread of comparable quality but with higher nutritional value can be made by partial substitution of wheat with triticale flour. Wholegrain flour from two Australian triticale cultivars and one wheat cultivar grown in a field trial (to exclude environmental effects) was compared for sourdough quality against a background of commercial wholemeal wheat flour. Partial substitution of wholegrain wheat flour for wholemeal wheat flour was included in the experiments to determine any effects of the type of wheat flour in the dough on sourdough bread quality.

## 2 MATERIALS AND METHODS

### 2.1 Grain and wholegrain flour samples

Grains of the triticale cultivars Goanna (G) and Hawkeye (H), as well as the bread wheat cultivar Scout (S), were harvested from a field trial run in 2019 at the I. A. Watson Grain Research Centre, a facility of the University of Sydney in Narrabri, NSW, Australia (Lat. ‐30.274249, Long. 149.809321). Triticale and wheat cultivars were grown on a gray vertosol in a randomized complete block design with two replicates. Each treatment plot was 1.8 × 3.8 m. Granulock Z® fertilizer (Incitec Pivot, Australia) was applied at 60 kg/ha at sowing, and trials were sprayed with pesticide and herbicide as required. The grains were harvested with a Haldrup C65 plot combine (Haldrup, Germany). Subsamples of harvested grain were cleaned with a Kimseed Vacuum Separator (Kimseed, Australia) to remove trash and broken grains. Grain was milled using a Newport Scientific Hammer Mill 6000 incorporating a 0.8‐mm screen (Newport Scientific, Australia) and sieved using a mesh size of 350 μm (N°55, Shangyu, China) to produce a wholegrain flour for the two triticale cultivars (GF and HF) and one wheat cultivar (SF). To clarify, the wholegrain flour was made by milling the grain directly into flour, whereas the commercial wholemeal flour would have been milled into white (endosperm) flour, germ, and bran, with the white flour and bran fractions recombined (and the germ excluded). Milled samples were used immediately for breadmaking. Commercial wholemeal wheat flour (White Wings brand) (WF) was obtained from an IGA supermarket in Sydney, Australia.

### 2.2 Wholegrain flour protein content

The total protein content of the milled flours GF, HF, and SF, plus the control WF, was determined using a nitrogen/protein Vario MACRO Cube analyzer (Frankfort, Germany). Flour samples were analyzed following the AOAC method 968.03. Thirty milligrams of each sample were prepared in 12 × 6‐mm pressed aluminum capsules. The samples were combusted with oxygen and nitrogen oxide and collected in a ballast tank. A helium gas carrier was used alongside the nitrogen oxide combustion gas, which was reduced to nitrogen. Water and carbon dioxide were then removed by passing the sample through magnesium perchlorate and sodium hydroxide on a silicate carrier. A thermal conductivity detector was utilized to measure the nitrogen content, using helium as a reference. The measured nitrogen content was converted to total protein content by applying a conversion factor of 5.81 for wheat and 5.7 for triticale (Fujihara et al., 2008; Wrigley et al., 2016). Measurements were performed in triplicate and expressed as mean values.

### 2.3 Flour substitution ratio and water content

Sourdough breads were made using 100% wholegrain triticale flour using each of the two triticale cultivars (GF and HF), 100% wholegrain wheat flour (SF), and 100% wholemeal wheat commercial flour as controls, each with a water content of 100 g/100 g flour. Additionally, to evaluate the effects of replacing wholemeal wheat flour with wholegrain triticale or wholegrain wheat flour on the texture and color of sourdough breadmaking, the following ratios of wholegrain milled flour to commercial wholemeal wheat flour were tested: 90:10 (*T*_1_), 80:20 (*T*_2_), 70:30 (*T*_3_), and 60:40 (*T*_4_), each with a water content of 100 g/100 g flour. The optimum flour ratio (of the four tested) was then selected for experiments to determine the effects of water content in the dough preparation in the following treatments: 70 g/100 g flour (*W*_1_), 80 g/100 g flour (*W*_2_), 90 g/100 g flour (*W*_3_), and 100 g/100 g flour (*W*_4_). Thus, the number of distinct loaves baked was 4 (100% GF, 100% HF, 100% SF, 100% WF) + [4 (*T*_1_, *T*_2_, *T*_3_, *T*_4_) × 3 (GF, HF, SF)] + [4 (*W*1, *W*2, *W*3, *W*4) × 3 (GF, HF, SF)] = 28 (each in triplicate; i.e., 84 loaves in total).

### 2.4 Dough and bread preparation

Sourdough bread was prepared using a commercial powdered starter that contained yeast, dextrose, *Lactococcus lactis* subsp. *cremoris*, *Lactococcus lactis* subsp. *lactis*, and *Lactococcus lactis* subsp. *lactis* bv. *diacetylactis* (Mad Millie Sourdough Culture (Starter), Naturemart). Breadmaking was based on a process requiring kneading. The dough was prepared by hand‐mixing the wholegrain triticale flour (180 g) or wholegrain wheat flour (180 g) with wholemeal wheat flour (20 g) and water (191 g) together for 2 min, then allowing the dough to incubate at room temperature for 45 min. The starter (4 g) and dough were combined slowly over 2 min. Salt (3 g) and malt (2 g) were added, and the dough was mixed slowly over 1 min and then rapidly until the dough was developed. The final dough (400 g) was allowed to bulk ferment for 2 h at room temperature and allowed to retard at 4°C for 12 h. Then the dough was proofed at room temperature for 3 h and baked in an oven (XECC‐0523‐E1R, Unox, Australia) at 150°C for 20 min. Three sourdough breads were prepared per treatment.

### 2.5 Dough pH, moisture content, water activity, and specific volume

Dough (D) pH was measured in a dough extract at room temperature using a pH meter (HI 9025, Hanna Instruments, Woonsocket, RI, USA) (Jayaram et al., 2013).

Moisture content for GH, HF, SF, and WF was measured using a gravimetric method. Samples (3 g) were weighed on a paper muffin liner and placed in an oven at 105°C for 3 h. Samples were analyzed in triplicate.

(1) $$\text{Moisture content}\;\left(\%\right)=\frac{H_{0}-H_{f}}{H_{0}}\times 100$$

where *H*_0_ represent the initial mass and *H*_*f*_ the final mass of the flour.

The water activity (*A*_*w*_) of the sourdough loaves (*L*) was measured using a water activity meter (Model AquaLab Pre; Decagon Devices, Pullman, Washington, USA). The specific volume of the sourdough bread was measured using the rapeseed displacement method (AACC Method 10–05.01) after cooling the loaves to room temperature. Measurements were performed in triplicate.

### 2.6 Dough and sourdough bread color

Color parameters were measured for the dough, as well as the sourdough bread crumb (C) and crust (CT) using a chromometer (Minolta Chroma Meter [Konika Minolta, USA]). Each sample was tested in triplicate for lightness (L*), redness/greenness (a*), and yellowness/blueness (b*). The brownness index (BI) and chroma (∆E) were derived using the following equations:

(2) $$x=\frac{a^{*}+1.75\times L^{*}}{5.645\times L^{*}+a^{*}-3.012\times b^{*}}$$

(3) $$\mathrm{BI}=100\times \left(x-0.31\right)/0.17$$

(4) $$\;\text{Chroma}=\sqrt{{\left(a^{*}\right)}^{2}+{\left(b^{*}\right)}^{2}}$$

### 2.7 Sourdough bread texture

Texture parameters were quantified for the crumb of the sourdough loaves for each treatment using a texture analyzer (TMS‐Touch; Food Technology Corporation, USA) fitted with a 500‐N load cell. Samples were cut into circular shapes (40 mm diameter × 20 mm height) using a stainless‐steel round cutter.

For the crumb, the middle portion of each loaf was used for texture determination. Samples were analyzed using a 75‐mm‐diameter probe, with a compression ratio of 30%, a pre‐test speed of 50 mm/min, a test speed of 72 mm/min, a distance of 13 mm, and a trigger force of 0.2 N. Hardness (HARD), springiness (SPRING), chewiness (CHEW), resilience (RES), cohesiveness (COH), and gumminess (GUM) were calculated using the TMS‐Touch software. The average values of eight samples were calculated.

### 2.8 Statistical analysis

Differences between all samples were evaluated using Analysis of Variance (ANOVA). Differences were calculated by Tukey's post hoc test with a significance level of *p* < .05. Pearson's coefficient correlations of two variables were performed with a significance level of *p* < .05. All statistical tests were conducted using SPSS‐Advanced Statistics 20 software (SPSS Inc, Chicago, IL).

## 3 RESULTS AND DISCUSSION

### 3.1 Protein content in triticale and wheat grain flour

The highest protein content (%) among the two triticale wholegrain flours was that of GF (12.76 ± 0.01, db), with HF having a substantially lower value (11.50 ± 0.01, db), while the wheat samples had the following protein contents: SF (12.40 ± 0.01, db) and WF (11.50 ± 0.01, db). Consistent with the results for the two triticale cultivars, Dennett et al. (2013) reported that the protein content in triticale grains is due partly to genotype, not only environment (e.g., soil available nitrogen). It was shown that the protein content of 11 triticale genotypes from a specific year of a field trial was 10.5%–14.6% (db), while with nine genotypes in another year of the same trial, the protein content was 11.8%–15.2% (db). Frás, Gołębiewska, Gołębiewski, Mańkowski, and Szecówka (2016) reported a protein content of triticale flour between 9.8 and 13.9% (db).

Stankowski et al. (2017) reported the influence of fertilizer type and nitrogen nutrition level on the baking and technological properties of spring triticale grain cv. Nagano grown in the Szczecin Lowland in north‐western Poland. Increasing amounts of nitrogen fertilizer increased the protein content up to a maximum of 12.7% in the triticale grains, a value consistent with those obtained here for GF.

### 3.2 Moisture content, *A*_*w*_, pH, and specific volume

Higher levels of moisture content (%) were observed for WF (13.03 ± 0.01), followed by HF (12.35 ± 0.02), SH (12.13 ± 0.01), and GF (12.01 ± 0.02) (*p* < .05). The ranges of A_w_ values for the sourdough loaves for which commercial wholemeal wheat flour was substituted with triticale or wheat wholegrain flour at different ratios were GL 0.90–0.95, HL 0.91–0.95, and SL 0.90–0.96. The values for the 100% wholegrain and 100% wholemeal flours were WL (0.92), GL (0.91), HL (0.91), and SL (0.92). When the water content was varied, the ranges of *A*_*w*_ values were GL 0.90–0.97, HL 0.90–0.96, and SL 0.90–0.97. *A*_*w*_ values increased slightly (*p* < .0001) with higher levels of replacement of wholemeal wheat flour, and, as expected, *A*_*w*_ increased when water content increased. pH ranged from 5.70 to 5.72; that is, no significant differences in pH were observed for dough samples among cultivars and treatments.

**FIGURE 1:**
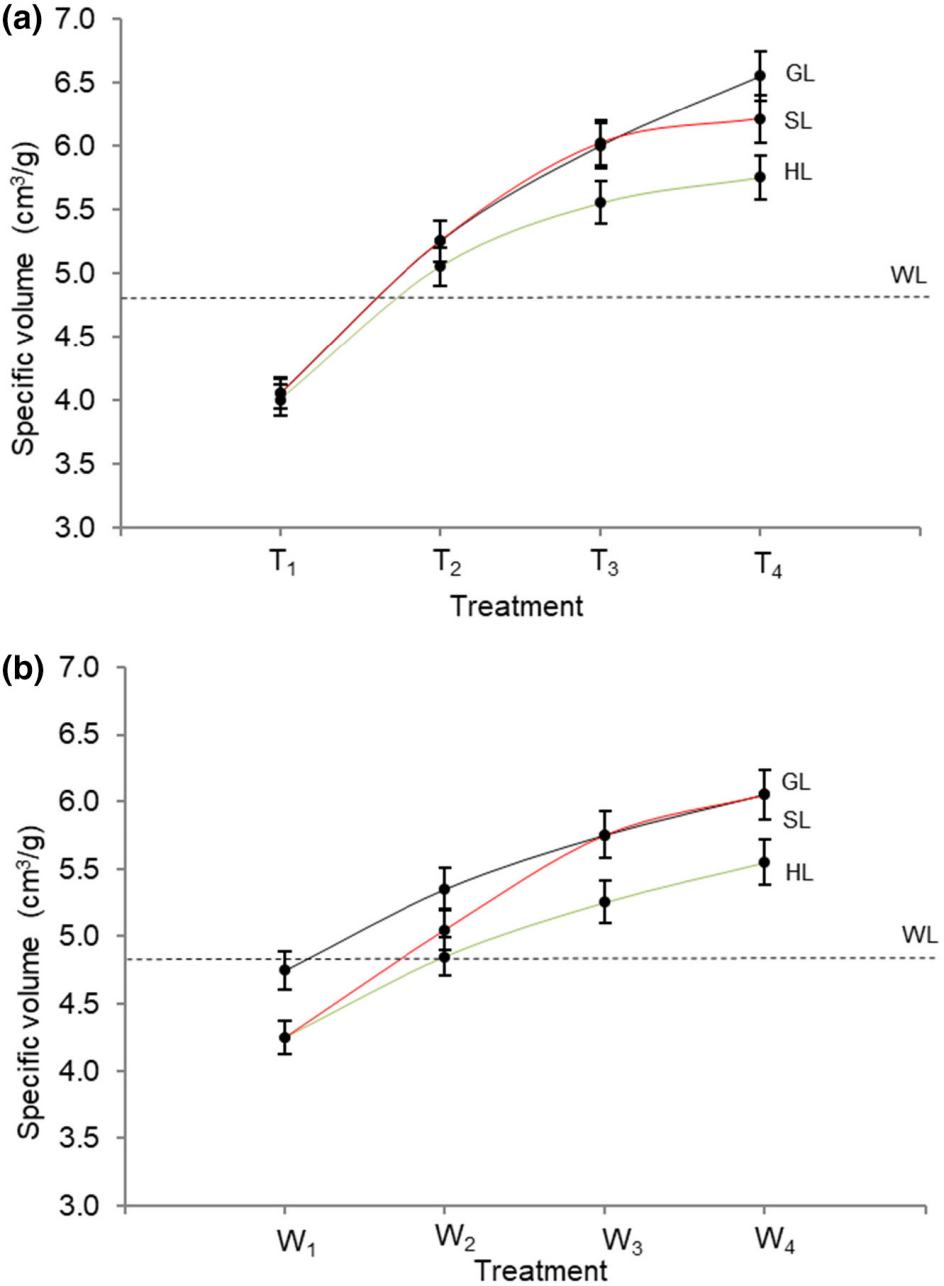
Specific volume of sourdough breads containing (a) different ratios of Goanna (triticale), Hawkeye (triticale), and Scout (wheat) wholegrain flour to commercial wholemeal wheat flour and (b) varying water content. GL, Goanna loaf; HL, Hawkeye loaf; SL, Scout loaf; *T*_1_, 90% wholegrain triticale flour and 10% wholemeal wheat flour; *T*_2_, 80% wholegrain triticale flour and 20% wholemeal wheat flour; *T*_3_, 70% wholegrain triticale flour and 30% wholemeal wheat flour; *T*_4_, 60% wholegrain triticale and 40% wholemeal wheat flour; *W*_1_, 70 g/100 g flour; *W*_2_, 80 g/100 g flour; *W*_3_, 90 g/100 g flour; *W*_4_, 100 g/100 g flour. For the experiment in panel b, the ratio of triticale or wholegrain wheat flour to commercial wholemeal wheat flour was held constant at 60%:40%. The dashed line represents 100% wholegrain wheat flour (WL).

The specific volumes of the loaves (in cm^3^/g) with different ratios of wholegrain triticale or wheat flour to commercial wholemeal wheat flour (Figure 1a) and varying the water content for SL, HL, and GL (Figure 1b) were determined. When the specific volumes of the samples with different flour ratios and varying water content were compared to the 100% wholemeal wheat flour control, results showed that specific volume increased from T_1_ to T_4_ as follows: for GL by 4.0%–6.5%, HL by 4.0%–5.7%, and SL by 4.0%–6.2%. For W_1_ to W_4_, the increases were as follows: GL by 4.7%–6.0%, HL by 4.2%–5.5%, and SL by 4.2%–6.0%. The specific volume for 100% wholemeal wheat flour and triticale wholegrain flour were as follows: WF (4.7%), GL (3.2%), SL (3.4%), and HL (2.9%). GL and SL had higher specific volumes than HL, which ranked the same as protein content. Loaves with 60% wholegrain Goanna triticale flour and 40% wholemeal wheat flour (i.e., *T*_4_) appeared to have a greater volume than loaves with Scout wheat or Hawkeye triticale also at *T*_4_ (Figure 1a). As expected, loaves made from 100% triticale wholegrain flour leavened very poorly, with specific volumes much lower than those of *T*_1_, which is why these data are not shown in Figure 1.

Tohver (2005) conducted baking tests with substitutions of 10%–70% wholegrain flour from 12 triticale genotypes for wholegrain wheat flour. Those breads with up to 50% triticale flour showed similar quality, including specific volume, as those made from wheat flour only. Incorporation of triticale flour over 50% substitution negatively affected quality parameters. The author made the point that the protein content of flour is extremely important because almost all flour properties, including gluten content, water absorption, mixing requirement, and specific volume of breads, are highly correlated with protein content. Doxastakis et al. (2002) also reported that the optimal substitution rate of triticale to wheat flour for bread is up to 50%.

Specific volume depends on flour protein quality, particularly levels of gliadin and glutenin in the gluten (Banu et al., 2011). These proteins are responsible for dough rheology, gas retention, and bread volume (Ortolan & Steel, 2017). Pattison et al. (2014) reported that among 17 modern triticale cultivars, the average white flour protein content was 16% lower in triticale compared to wheat, despite similar wholegrain protein contents, suggesting that triticale stores a lower proportion of protein in the endosperm.

Our results showed that using higher substitution levels of wholegrain triticale or wheat flour for wholemeal wheat flour, as well as higher levels of water in the dough, increased specific volume. The addition of more water to a dough (up to a point) is expected to create a stronger network between proteins, allowing a major retention of gas when leavening with sourdough starter, which will increase the volume in the bread (Biel et al., 2020). GL showed a higher specific volume compared to HL, which may have been due to the higher protein content of GL.

Pearson's correlation (*p* < .05) with two factors, specific volume (cm^3^/g) and A_w_, for samples with different substitution levels of wholegrain GL, HL, and SL flour and varying water content showed a positive correlation between specific volume (cm^3^/g) and *A*_*w*_. High *r* values were observed among all the samples and treatments (*T*_1–4_ and *W*_1–4_): GL (*r* = 0.991), HL (*r* = .998), SL (*r* = .997), GL (*r* = .989), HL (*r* = .989), and SL (*r* = .991).

### 3.3 Color parameters

Bread color is considered an important visual factor, influencing consumer preferences and purchasing decisions. Analysis showed the influence of different substitution rates of triticale and varying water content on L*, a*, b*, BI, and chroma values for dough, crust, and crumb color (Tables [Link], [Link]).

L* and b* dough values slightly increased from *T*_1_ to *T*_4_, and a* and BI decreased from *T*_1_ to *T*_4_. For the crust, L* decreased from *T*_1_ to *T*_4_, and a*, b*, and BI increased from *T*_1_ to *T*_4_ for GCT, HCT, and SCT. For the crumb, L* and b* increased from *T*_1_ to *T*_4_, while a* and BI decreased from *T*_1_ to *T*_4_ for GC, HC, and SC.

Color parameters for the dough, crumb, and crust of the sourdough bread were also influenced by water content. For the dough, L* values increased from *W*_1_ to *W*_4_; a*, b*, and BI decreased from *W*_1_ to *W*_4_ for GD, HD, and SD. For the crust, L*, b*, and BI decreased from *W*_1_ to *W*_4_, and a* increased from *W*_1_ to *W*_4_ for GCT, HCT, and SCT. For the crumb, L* increased from *W*_1_ to *W*_4_, and a*, b*, and BI decreased from *W*_1_ to *W*_4_ for GC, HC, and SC.

**FIGURE 2:**
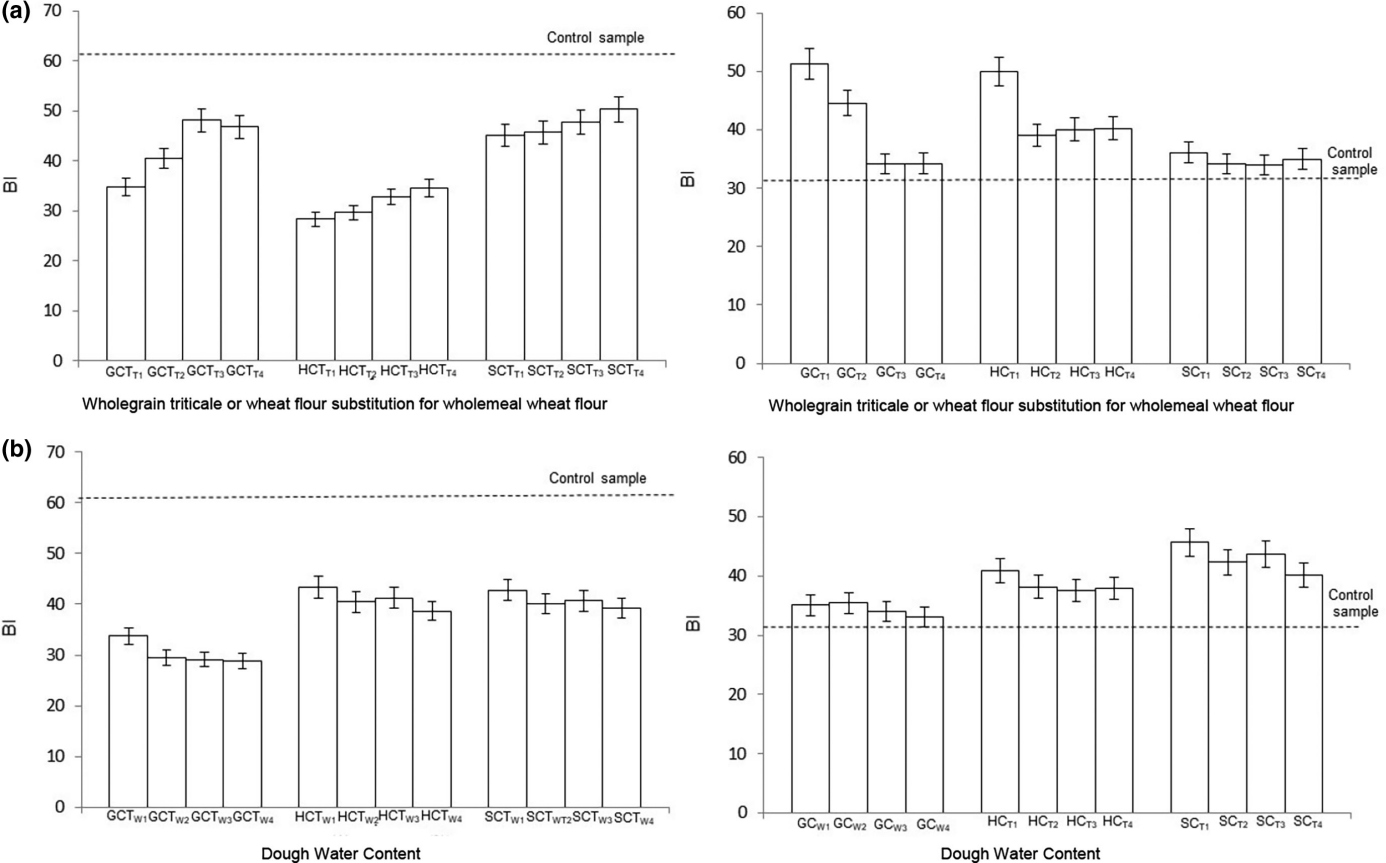
Brownness index (BI) of Goanna (triticale), Hawkeye (triticale), and Scout (wheat) sourdough bread crust and crumb containing (a) different ratios of wholegrain flour to commercial wholemeal wheat flour and (b) varying water content (percentage of the maximum water content tested). GC, Goanna crumb; GCT, Goanna crust; HC, Hawkeye crumb; HCT, Hawkeye crust; SC, Scout crumb; SCT, Scout crust; *W*_1_, 70% water; *W*_2_, 80% water; *W*_3_, 90% water; *W*_*4*_, 100% water; *T*_1_, 90% wholegrain triticale flour and 10% wholemeal wheat flour; *T*_2_, 80% wholemeal triticale and 20% wholemeal wheat flour; *T*_3_, 70% wholemeal triticale and 30% wholegrain wheat flour; *T*_4_, 60% wholemeal triticale and 40% wholemeal wheat flour. For comparison, the BI of the control crust and crumb (wholemeal sourdough wheat bread) is depicted as a dotted horizontal line.

Figure 2 shows BI for the crumb and crust for the various cultivars and treatments. For the crust, BI values for the loaves containing wholegrain triticale or wheat flour were below the control sample, whereas for the crumb, the BI values were above the control sample. In traditional wholegrain triticale bread, increasing amounts of triticale in the flour blend have been shown to have a significant effect on the color of the crust (Sheikholeslami et al., 2020). L* values decreased when the proportion of triticale flour was increased, leading to a darker crust. a* values increased when wholegrain triticale content was increased, while b* values increased after increasing the triticale flour proportion. The crumb color was also darker in samples containing higher amounts of wholegrain triticale flour.

Pycia et al. (2018) also reported that L* values for triticale bread fell as the proportion of triticale increased. The lower values of L* were thought to result from the substantial content of monosaccharides in the dough, which are a substrate for the Maillard reaction during baking at high temperatures.

The brownness index decreases in crust and crumb when the water content of bread increases; water content plays an important role in the Maillard reaction and caramelization, which lead to changes in color during the baking process (Sahin et al., 2020). The addition of water leads to a decrease in the extent of the browning reaction because of the dilution of the browning compounds (Fatemi, 2003). Zain et al. (2023) reported that, in wholemeal bread, the brownness index in the crust decreased when higher levels of water (80%–105%) were added during breadmaking. L* values increased as water addition increased, indicating increased crust brightness. This suggests that the color parameters were less affected at higher water amounts. The author stated that the addition of 80% water improves color parameters in breadmaking.

Changes in color are also due to reactions occurring at high temperatures, such as protein denaturation, enzyme inactivation, evaporation of moisture, caramelization, and the Maillard reaction (Dennett & Trethowan, 2013; Mezaize et al., 2009; Navarro‐Contreras et al., 2014). Darker breads associated with increasing levels of triticale flour can be due to pigments in the triticale flour and a lower content of water (Sheikholeslami et al., 2020).

### 3.4 Texture parameters

Statistical analysis (Table 1) showed the influence of different substitution levels of wholegrain triticale or wheat flour for wholemeal wheat flour, as well as varying water content, on texture parameters (HARD, COH, GUM, SPRING, CHEW, and RES). HARD, CHEW, and GUM, which are important factors for assessing bread texture, decreased from *T*_1_ to *T*_4_ for GC, HC and SC for both treatments. Texture values for WC: HARD (5.49), COH (0.64), GUM (2.92), SPRING (0.21), CHEW (3.88), and RES (0.14); GC: HARD (7.99), COH (0.59), GUM (4.81), SPRING (0.42), CHEW (8.71), and RES (0.21); HC: HARD (19.41), COH (0.63), GUM (11.1), SPRING (0.17), CHEW (10.70), and RES (0.22), and SC: HARD (7.43), COH (0.91), GUM (5.24), SPRING (0.22), CHEW (6.75), and RES (0.41).

**TABLE 1:** Texture parameters for sourdough bread crumb containing different ratios of Goanna (triticale), Hawkeye (triticale), or Scout (wheat) wholegrain flour to commercial wholemeal wheat flour and varying dough water content.

Sample	Texture parameter					
	HARD	COH	GUM	SPRING	CHEW	RES
GC_*T*1_	6.93 ± 0.36^a^	0.48 ± 0.01^b^	3.49 ± 0.30^a^	0.38 ± 0.02^b^	7.27 ± 0.16^a^	0.20 ± 0.04^b^
GC_*T*2_	6.86 ± 0.16^a^	0.43 ± 0.02^b^	3.25 ± 0.34^ab^	0.38 ± 0.03^b^	7.23 ± 0.06^a^	0.17 ± 0.01^b^
GC_*T*3_	6.99 ± 0.54^a^	0.54 ± 0.02^a^	2.81 ± 0.49^bc^	0.40 ± 0.02^b^	7.49 ± 0.03^a^	0.29 ± 0.02^a^
GC_*T*4_	5.90 ± 0.26^b^	0.56 ± 0.02^a^	2.43 ± 0.14^c^	0.45 ± 0.14^a^	3.28 ± 0.23^b^	0.29 ± 0.03^a^
*p*‐Value	.0001	.0001	.0001	.0001	.0001	.0001
HC_*T*1_	18.94 ± 0.53^a^	0.52 ± 0.03^a^	10.00 ± 0.35^a^	0.18 ± 0.03^b^	9.90 ± 0.87^a^	0.19 ± 0.01^b^
HC_*T*2_	13.20 ± 0.43^b^	0.51 ± 0.04^ab^	7.06 ± 0.08^b^	0.16 ± 0.02^b^	7.80 ± 0.59^b^	0.19 ± 0.02^b^
HC_*T*3_	9.75 ± 0.64^c^	0.49 ± 0.02^bc^	4.69 ± 0.05^c^	0.17 ± 0.02^b^	6.74 ± 0.43^c^	0.19 ± 0.01^b^
HC_*T*4_	8.16 ± 0.48^d^	0.47 ± 0.02^c^	4.08 ± 0.11^d^	0.39 ± 0.02^a^	4.18 ± 0.40^d^	0.21 ± 0.01^a^
*p*‐Value	.0001	NS	.0001	.0001	.0001	NS
SC_*T*1_	6.23 ± 0.50^a^	0.87 ± 0.05^a^	4.94 ± 0.04^a^	0.19 ± 0.05^bc^	5.25 ± 0.32^a^	0.38 ± 0.04^ab^
SC_*T*2_	6.14 ± 0.07^a^	0.79 ± 0.04^b^	4.30 ± 0.46^b^	0.23 ± 0.02^b^	4.00 ± 0.49^b^	0.33 ± 0.02^b^
SC_*T*3_	5.91 ± 0.90^b^	0.91 ± 0.03^a^	4.03 ± 0.35^b^	0.17 ± 0.03^c^	3.95 ± 0.40^b^	0.42 ± 0.05^a^
SC_*T*4_	5.90 ± 0.56^b^	0.73 ± 0.08^b^	3.34 ± 0.22^c^	0.31 ± 0.01^a^	2.83 ± 0.18^c^	0.21 ± 0.04^c^
*p*‐Value	.0001	.0001	.0001	.0001	.0001	.0001
GC_*W*1_	9.89 ± 0.63^a^	0.48 ± 0.04^b^	4.64 ± 0.19^a^	0.10 ± 0.00^d^	3.40 ± 0.07^a^	0.10 ± 0.00^a^
GC_*W*2_	9.24 ± 0.33^a^	0.44 ± 0.01^b^	4.13 ± 0.27^b^	0.19 ± 0.00^c^	3.66 ± 0.04^a^	0.06 ± 0.01^b^
GC_*W*3_	6.98 ± 0.17^b^	0.44 ± 0.03^b^	3.15 ± 0.31^c^	0.31 ± 0.02^a^	3.50 ± 0.19^a^	0.07 ± 0.01^b^
GC_*W*4_	5.67 ± 0.38^c^	0.54 ± 0.02^a^	3.07 ± 0.28^c^	0.25 ± 0.01^b^	1.17 ± 0.12^b^	0.09 ± 0.00^a^
*p*‐Value	.0001	.0001	.0001	.0001	.0001	.0001
HC_*W*1_	13.48 ± 0.93^a^	0.57 ± 0.02^a^	10.18 ± 0.69^a^	0.24 ± 0.01^b^	15.86 ± 0.97^a^	0.05 ± 0.01^c^
HC_*W*2_	10.12 ± 0.77^b^	0.46 ± 0.02^b^	5.00 ± 0.37^b^	0.14 ± 0.02^c^	8.86 ± 0.78^b^	0.19 ± 0.02^a^
HC_*W*3_	9.70 ± 0.21^b^	0.46 ± 0.03^b^	4.35 ± 0.50^b^	0.32 ± 0.02^a^	7.57 ± 0.35^c^	0.10 ± 0.02^b^
HC_*W*4_	9.60 ± 0.23^b^	0.45 ± 0.03^b^	4.25 ± 0.57^b^	0.33 ± 0.04^a^	3.46 ± 0.41^d^	0.10 ± 0.01^b^
*p*‐Value	.0001	.0001	.0001	.0001	.0001	.0001
SC_*W*1_	9.43 ± 0.56^a^	0.72 ± 0.03^c^	7.71 ± 0.83^a^	0.11 ± 0.04^c^	9.06 ± 0.54^a^	0.39 ± 0.01^a^
SC_*W*2_	6.85 ± 0.67^b^	0.87 ± 0.09^a^	6.83 ± 0.26^b^	0.25 ± 0.02^a^	6.41 ± 0.38^b^	0.33 ± 0.02^b^
SC_*W*3_	6.72 ± 0.44^b^	0.82 ± 0.02^b^	5.87 ± 0.50^c^	0.09 ± 0.09^c^	4.73 ± 0.66^c^	0.13 ± 0.01^c^
SC_*W*4_	5.91 ± 0.46^c^	0.53 ± 0.02^d^	2.94 ± 0.24^d^	0.20 ± 0.02^b^	3.29 ± 0.18^d^	0.10 ± 0.01^c^
*p*‐Value	.0001	.0001	.0001	.0001	.0001	.0001

*Note*: Means denoted by different letters in the columns indicate a significant difference between samples (*p* < .05) (Tukey's test). Goanna crumb (GC), Hawkeye crumb (HC), Scout crumb (SC), 90% wholegrain triticale or wheat flour and 10% wholemeal wheat flour (*T*_1_), 80% wholegrain triticale or wheat flour and 20% wholemeal wheat flour (*T*_2_), 70% wholegrain triticale or wheat flour and 30% wholemeal wheat flour (*T*_3_), 60% wholegrain triticale or wheat flour and 40% wholemeal wheat flour (*T*_4_), 70 g water/100 g flour (*W*_1_), 80 g water/100 g flour (*W*_2_), 90 g water/100 g flour (*W*_3_), 100 g water/100 g flour (*W*_4_).

Abbreviations: CHEW, chewiness; COH, cohesiveness; GUM, Gumminess; HARD, hardness; NS, not significant; RES, resilience; SPRING, springiness.

GC and SC at *T*_4_, as well as GC and SC at *W*_4_, had similar HARD values to the wholemeal wheat control. All other treatments showed greater hardness when compared to the control, with cultivar Hawkeye having the highest HARD value. This behavior might be related to the less extensive gluten matrix and greater fiber content in Hawkeye triticale flour compared to wholemeal wheat flour (Burešová et al., 2017; Patil et al., 2016). It can also be associated with the ability of triticale to absorb less moisture than wheat (Naeem et al., 2002) or the lower ability of starch to react with water (Huang et al., 2010).

In general, our results showed that, in terms of sourdough bread texture, Goanna triticale was superior to Hawkeye and resembled Scout wheat. Changes in texture, especially in hardness, can be associated with moisture content, strength of the gluten matrix, fiber content, and starch in the blended flour (Cauvain, 2004; Dvořakova et al., 2012). The effects of adding different amounts of wholegrain triticale flour as a replacement for wholemeal wheat flour and varying the amount of water allowed us to create sourdough bread with similar properties to traditional wholemeal wheat sourdough bread.

## 4 CONCLUSION

A blend of 60% wholemeal triticale flour and 40% wholemeal wheat flour with 100 g water/100 g flour in the dough preparation created sourdough bread with the most favorable specific volume, texture, and color among the treatments tested. Replacement of wholemeal wheat flour with wholegrain triticale flour at a 60:40 substitution level is a good option in sourdough breadmaking as it allows a bread to be made with similar quality to a traditional wholemeal wheat sourdough bread (or to a sourdough bread made with wholegrain wheat flour and wholemeal wheat flour at 60:40 substitution level). From a nutritional perspective, wholegrain triticale flour is richer in vitamins, minerals, phosphorus, and essential amino acids when compared to wholemeal wheat flour, and thus substitution should be encouraged. Among the two Australian triticale cultivars tested, Goanna was superior to Hawkeye.

Future studies will include nutritional and functional properties of the flour and sourdough bread samples, such as amino acid profiles, gluten quantification, and rheology, to better understand the effects of including wholegrain triticale flour in sourdough bread making.

## AUTHOR CONTRIBUTIONS

**Valeria Messina:** Formal analysis (lead); investigation (equal); methodology (equal); supervision (supporting); writing – original draft (equal). **Jason Cano:** Investigation (equal); writing – original draft (equal). **Anthony Silvio:** Investigation (supporting); methodology (equal); resources (equal); supervision (supporting). **Angela L. Pattison:** Conceptualization (equal); formal analysis (supporting); writing – review and editing (equal). **Thomas H. Roberts:** Conceptualization (equal); formal analysis (supporting); project administration (lead); supervision (equal); writing – review and editing (equal).

## CONFLICT OF INTEREST STATEMENT

The authors declare that they have no conflict of interest.

## Supporting information


Table S1.

Table S2.

Table S3.

## Data Availability

The data that support the findings of this study are available on request from the corresponding author.

## References

[fsn34050-bib-0001] Alfonzo, A. , Urso, V. , Corona, O. , Francesca, N. , Amato, G. , Settanni, L. , & Di Miceli, G. (2016). Development of a method for the direct fermentation of semolina by selected sourdough lactic acid bacteria. International Journal of Food Microbiology, 239, 65–78. 10.1016/j.ijfoodmicro.2016.06.027 27374130

[fsn34050-bib-0002] Arora, K. , Ameur, H. , Polo, A. , Di Cagno, R. , Giuseppe, R. , & Gobbetti, M. (2021). Thirty years of knowledge on sourdough fermentation: A systematic review. Trends in Food Science & Technology, 108, 71–83. 10.1016/j.tifs.2020.12.008

[fsn34050-bib-0003] Banu, I. , Vasilean, I. , Barbu, V. , & Iancu, C. (2011). The effect of some technological factors on the rye sourdough bread. Scientific Study and Research: Chemistry and Chemical Engineering, 12(2), 197–202.

[fsn34050-bib-0004] Biel, W. , Kazimierska, K. , & Bashutska, U. (2020). Nutritional value of wheat, triticale, barley and oat grains. Acta Scientiarum Polonorum Zootechnica, 19(2), 19–28. 10.21005/asp.2020.19.2.03

[fsn34050-bib-0005] Burešová, I. , Tokár, M. , Mareček, J. , Hřivna, L. , Faměra, O. , & Šottníková, V. (2017). The comparison of the effect of added amaranth, buckwheat, chickpea, corn, millet and quinoa flour on rice dough rheological characteristics, textural and sensory quality of bread. Journal of Cereal Science, 75, 158–164. 10.1016/j.jcs.2017.04.004

[fsn34050-bib-0006] Cauvain, S. P. (2004). Improving the texture of bread. In D. Kilcast (Ed.), (Ed.) Texture in food (pp. 432–450). Woodhead Publishing. 10.1533/978185538362.3.432

[fsn34050-bib-0007] Chavan, R. S. , & Chavan, S. R. (2011). Sourdough technology‐a traditional way for wholesome foods: A review. Comprehensive Reviews in Food Science and Food Safety, 10(3), 169–182. 10.1111/j.1541-4337.2011.00148.x

[fsn34050-bib-0008] Couch, G. W. (2016). Effect of sourdough fermentation parameters on bread properties . Master of Science thesis, Clemson University https://tigerprints.clemson.edu/all_theses/2581/

[fsn34050-bib-0009] D'Alessandro, A. , & De Pergola, G. (2014). Mediterranean diet pyramid: A proposal for Italian people. Nutrients, 6(10), 4302–4316. 10.3390/nu6104302 25325250 PMC4210917

[fsn34050-bib-0010] De Vuyst, L. , Harth, H. , Kerrebroeck, S. , & Leroy, F. (2016). Yeast diversity of sourdoughs and associated metabolic properties and functionalities. International Journal of Food Microbiology, 239, 26–34. 10.1016/j.ijfoodmicro.2016.07.018 27470533

[fsn34050-bib-0011] Deleu, L. J. , Lemmens, E. , Redant, L. , & Delcour, J. A. (2020). The major constituents of rye (*Secale cereale* L.) flour and their role in the production of rye bread, a food product to which a multitude of health aspects are ascribed. Cereal Chemistry, 97(4), 739–754. 10.1002/cche.10306

[fsn34050-bib-0012] Demirkesen‐Bicak, H. , Arici, M. , Yaman, M. , Karasu, S. , & Sagdic, O. (2021). Effect of different fermentation condition on estimated glycemic index, in vitro starch digestibility, and textural and sensory properties of sourdough bread. Food, 10(3), 514. 10.3390/foods10030514 PMC8000543 33804465

[fsn34050-bib-0013] Dennett, A. , & Trethowan, R. M. (2013). Milling efficiency of triticale grain for commercial flour production. Journal of Cereal Science, 57(3), 527–530. 10.1016/j.jcs.2013.03.002

[fsn34050-bib-0014] Dennett, A. L. , Cooper, K. V. , & Trethowan, R. M. (2013). The genotypic and phenotypic interaction of wheat and rye storage proteins in primary triticale. Euphytica, 194(2), 235–242. 10.1007/s10681-013-0950-y

[fsn34050-bib-0015] Doxastakis, G. , Zafiriadis, J. , Irakli, M. , Marlani, H. , & Tananaki, C. (2002). Lupin, soya and triticale addition to wheat flour doughs and their effect on rheological properties. Food Chemistry, 77(2), 219–227. 10.1016/S0308-8146(01)00362-4

[fsn34050-bib-0016] Dvořakova, P. , Burešova, I. , & Kračmar, S. (2012). Textual properties of bread formulations based on buckwheat and rye flour. Acta Universitatis Agriculturae et Silviculturae Mendelianae Brunensis, 60(5), 61–68.

[fsn34050-bib-0017] Fatemi, H. (2003). Food chemistry (2nd ed., p. 481). Sahami Company of Enteshar.

[fsn34050-bib-0018] Fraberger, V. , Unger, C. , Kummer, C. , & Domig, K. J. (2020). Insights into microbial diversity of traditional Austrian sourdough. LWT–Food Science and Technology, 127, 109358. 10.1016/j.lwt.2020.109358

[fsn34050-bib-0019] Fraś, A. , Gołębiewski, D. , Gołębiewska, K. , Mańkowski, D. , Boros, D. , & Gzowska, M. (2016). Triticale‐oat bread as a new product rich in bioactive and nutrient components. Journal of Cereal Science, 82, 146–154. 10.1016/j.jcs.2018.05.001

[fsn34050-bib-0020] Fraś, K. , Gołębiewska, D. , Gołębiewski, D. , Mańkowski, D. , & Szecówka, P. (2016). Variability in the chemical composition of triticale grain, flour and bread. Journal of Cereal Science, 71, 66–72. 10.1016/j.jcs.2018.05.001

[fsn34050-bib-0021] Fujihara, S. , Sasaki, H. , Aoyagi, Y. , & Sugahara, T. (2008). Nitrogen‐to‐protein conversion factors for some cereal products in Japan. Journal of Food Science, 73(3), C204–C209. 10.1111/j.1750-3841.2008.00665.x 18387100

[fsn34050-bib-0022] Galoburda, R. , Straumite, E. , Sabovics, M. , & Kruma, Z. (2020). Dynamics of volatile compounds in triticale bread with sourdough: From flour to bread. Food, 9(12), 1837. 10.3390/foods9121837 PMC7763431 33321806

[fsn34050-bib-0023] Gänzle, M. G. , & Zheng, J. (2019). Lifestyles of sourdough lactobacilli – Do they matter for microbial ecology and bread quality. International Journal of Food Microbiology, 302, 15–23. 10.1016/j.ijfoodmicro.2018.08.019 30172443

[fsn34050-bib-0024] Gobbetti, M. , De Angelis, M. , Di Cagno, R. , Calasso, M. , Archetti, G. , & Rizzello, C. G. (2019). Novel insights on the functional/nutritional features of the sourdough fermentation. International Journal of Food Microbiology, 302, 103–113. 10.1016/j.ijfoodmicro.2018.05.018 29801967

[fsn34050-bib-0025] Goryanina, T. A. (2020). Production of bread for healthy food from winter triticale, wheat and rye. Scientific achievements of the third millennium. Collection of scientific papers on materials XI international scientific conference. Part 1. International united Academy of Sciences. Chicago 10.18411/scienceconf-05-2020-04

[fsn34050-bib-0026] Huang, C. , Lai, P. , Chen, I. H. , Liu, Y. , & Wang, C. (2010). Effects of mucilage on the thermal and pasting properties of yam, taro, and sweet potato starches. LWT‐ Food Science and Technology, 43(6), 849–855. 10.1016/j.lwt.2009.11.009

[fsn34050-bib-0027] Järvan, M. , Lukme, L. , Adamson, A. , & Akk, A. (2017). Responses of wheat yield, quality and bread‐making properties on the sulphur fertilization. Acta Agriculturae Scandinavica Section B Soil and Plant Science, 67(5), 444–452. 10.1080/09064710.2017.1293725

[fsn34050-bib-0028] Jayaram, V. B. , Cuyvers, S. , Lagrain, B. , Verstrepen, K. J. , Delcour, J. A. , & Courtin, C. M. (2013). Mapping of *Saccharomyces cerevisiae* metabolites in fermenting wheat straight‐dough reveals succinic acid as pH‐determining factor. Food Chemistry, 136(2), 301–308. 10.1016/j.foodchem.2012.08.039 23122062

[fsn34050-bib-0029] Ma, S. , Wang, Z. , Guo, X. , Wang, F. , Huang, J. , Sun, B. , & Wang, X. (2021). Sourdough improves the quality of whole‐wheat flour products: Mechanisms and challenges—A review. Food Chemistry, 360, 130038. 10.1016/j.foodchem.2021.130038 34020364

[fsn34050-bib-0030] McGoverin, G. , Snyders, F. , Muller, N. , Botes, W. , Fox, G. , & Manley, M. (2011). A review of triticale uses and the effect of growth environment on grain quality. Journal of the Science of Food and Agriculture, 91(7), 1155–1165. 10.1002/jsfa.4338 21433010

[fsn34050-bib-0031] Mezaize, S. , Chevallier, S. , Le Bail, A. , & De Lamballerie, M. (2009). Optimization of gluten‐free formulations for French‐style breads. Food Science, 74(3), 140–146. 10.1111/j.1750-3841.2009.01096.x 19397719

[fsn34050-bib-0032] Mihhalevski, A. , Heinmaa, I. , Traksmaa, R. , Pehk, T. , Mere, A. , & Paalme, T. (2012). Structural changes of starch during baking and staling of Rye bread. Journal of Agricultural and Food Chemistry, 60(34), 8492–8500. 10.1021/jf3021877 22889064

[fsn34050-bib-0033] Naeem, H. , Darvey, N. , Gras, P. , & MacRitchie, F. (2002). Mixing properties, baking potential, and functionality changes in storage proteins during dough development of triticale‐wheat flour blends. Cereal Chemistry, 79(3), 332–339. 10.1094/CCHEM.2002.79.3.332

[fsn34050-bib-0034] Navarro‐Contreras, L. , Chaires‐González, C. , Rosas‐Burgos, E. , Borboa, J. , Flores, F. , Wong‐Corral, M. , & Cortez‐Rocha, O. (2014). Comparison of protein and starch content of substituted and complete triticales (X *Triticosecale* Wittmack): Contribution to functional properties. International Journal of Food Properties, 17, 421–432. 10.1080/10942912.2011.642440

[fsn34050-bib-0035] Ortolan, F. , & Steel, C. (2017). Protein characteristics that affect the quality of vital wheat gluten to be used in baking: A review. Comprehensive Reviews in Food Science and Food Safety, 16(3), 369–381. 10.1111/1541-4337.12259 33371555

[fsn34050-bib-0036] Papadimitriou, K. , Zoumpopoulou, G. , Georgalaki, M. , Alexandraki, V. , Kazou, M. , Anastasiou, R. , & Tsakalidou, E. (2019). Sourdough bread. In Innovations in traditional foods (pp. 127–158). Woodhead Publishing. 10.1016/B978-0-12-814887-7.00006-X

[fsn34050-bib-0037] Patil, S. , Rudra, S. , Varghese, E. , & Kaur, C. (2016). Effect of extruded finger millet (*Eleusine coracan* L.) on textural properties and sensory acceptability of composite bread. Food Bioscience, 14, 62–69. 10.1016/j.fbio.2016.04.001

[fsn34050-bib-0038] Pattison, A. L. , Appelbee, M. , & Trethowan, R. M. (2014). Characteristics of modern triticale quality: Glutenin and Secalin subunit composition and Mixograph properties. Journal of Agricultural and Food Chemistry, 62(21), 4924–4931. 10.1021/jf405138w 24792750

[fsn34050-bib-0039] Petrova, P. , & Petrov, K. (2020). Lactic acid fermentation of cereals and pseudocereals: Ancient nutritional biotechnologies with modern applications. Nutrients, 12(4), 1118. 10.3390/nu12041118 32316499 PMC7230154

[fsn34050-bib-0040] Plessas, S. (2021). Innovations in sourdough bread making. Fermentation, 7(1), 29. 10.3390/fermentation7010029

[fsn34050-bib-0041] Pycia, K. , Jaworska, G. , Telega, J. , Sudoł, I. , & Kuźniar, P. (2018). Effect of adding potato maltodextrins on baking properties of triticale flour and quality of bread. LWT‐ Food Science and Technology, 96, 199–204. 10.1016/j.lwt.2018.05.039

[fsn34050-bib-0042] Sahin, A. W. , Wiertz, J. , & Arendt, E. K. (2020). Evaluation of a new method to determine the water addition level in gluten free bread systems. Journal of Cereal Science, 93, 1–8. 10.1016/j.jcs.2020.102971

[fsn34050-bib-0043] Sheikholeslami, Z. , Mahfouzi, M. , Karimi, M. , Hejrani, T. , Ghiafehdavoodi, M. , & Ghodsi, M. (2020). Evaluating the traditional bread properties with new formula: Affected by triticale and cress seed gum. Food Science and Technology International, 27, 1082013220961777. 10.1177/1082013220961777 33019815

[fsn34050-bib-0044] Siepmann, F. B. , Ripari, V. , Waszczynskyj, N. , & Spier, M. R. (2018). Overview of sourdough technology: From production to marketing. Food and Bioprocess Technology, 11, 242–270. 10.1007/s11947-017-1968-2

[fsn34050-bib-0045] Silva, A. , Ramosa, M. , Ribeiro Júnior, W. , Rodrigues de Alencara, E. , Carvalho da Silva, P. , de Lima, C. , Vinson, C. , & Vanderlei Silva, M. (2020). Water stress alters physical and chemical quality in grains of common bean, triticale and wheat. Agricultural Water Management, 231, 16023. 10.1016/j.agwat.2020.106023

[fsn34050-bib-0046] Stankowski, S. , Sobolewska, M. , Jaroszewska, A. , & Michalska, B. (2017). Impact of form and dose of nitrogen fertilizers on the technological value of spring triticale (x *Triticosecale* Wittm. ex A. Camus). Folia Pomeranae Universitatis Technologiae Stetinensis, seria Agricultura, Alimentaria, Piscaria et Zootechnica, 336(43), 167–178.

[fsn34050-bib-0047] Tohver, M. (2005). Quality of triticale cultivars suitable for growing and bread‐making in northern conditions. Food Chemistry, 89(1), 125–132. 10.1016/j.foodchem.2004.01.079

[fsn34050-bib-0048] Ventimiglia, G. , Alfonzo, A. , Galluzzo, P. , Corona, O. , Francesca, N. , Caracappa, S. , Moschetti, G. , & Settanni, L. (2015). Codominance of Lactobacillus plantarum and obligate heterofermentative lactic acid bacteria during sourdough fermentation. Food Microbiology, 51, 57–68. 10.1016/j.fm.2015.04.011 26187828

[fsn34050-bib-0049] Wang, P. , Tao, H. , Jin, Z. , & Xu, X. (2016). Impact of water extractable arabinoxylan from rye bran on the frozen steamed bread dough quality. Food Chemistry, 200, 117–124. 10.1016/j.foodchem.2016.01.027 26830568

[fsn34050-bib-0050] Wang, S. , Thomas, K. C. , Sosulski, K. , Ingledew, W. M. , & Sosulski, F. W. (1999). Grain pearling and very high gravity (VHG) fermentation technologies for fuel alcohol production from rye and triticale. Process Biochemistry, 34(5), 421–428. 10.1016/s0032-9592(98)00097-1

[fsn34050-bib-0051] Wang, Z. , Li, Y. , Yang, Y. , Liu, X. , Qin, H. , Dong, Z. , Zheng, S. , Zhang, K. , & Wang, D. (2017). New insight into the function of wheat glutenin proteins as investigated with two series of genetic mutants. Scientific Reports, 7, 3428. 10.1038/s41598-017-03393-6 28611351 PMC5469833

[fsn34050-bib-0052] Wrigley, C. , Batey, I. , & Miskelly, D. (2016). Cereal grains: Assessing and managing quality (p. 830). Second Edition Woodhead Publishing.

[fsn34050-bib-0053] Wu, S. , Peng, Y. , Xi, J. , Zhao, Q. , Xu, D. , Jin, Z. , & Xu, X. (2022). Effect of sourdough fermented with corn oil and lactic acid bacteria on bread flavor. LWT‐ Food Science and Technology, 155, 112935. 10.1016/j.lwt.2021.112935

[fsn34050-bib-0054] Yildrime, R. M. , & Arici, M. (2019). Effect of the fermentation temperature on the degradation of phytic acid in whole‐wheat sourdough bread. LWT‐ Food Science and Technology, 112, 108224. 10.1016/j.lwt.2019.05.122

[fsn34050-bib-0055] Zain, N. , Ghani, M. , & Kasim, Z. (2023). Effects of water addition level on physical properties, rheological profile and sensory evaluation of gluten‐free bread: A preliminary approach. Sains Malaysiana, 52, 487–499. 10.17576/jsm-2023-5202-13

[fsn34050-bib-0056] Zhu, F. (2018). Triticale: Nutritional composition and food uses. Food Chemistry, 241, 468–479. 10.1016/j.foodchem.2017.09.00 28958555

[fsn34050-bib-0057] Zlateva, D. , Stefanova, D. , & Ninova‐Nicolova, N. (2017). Study on the development of lactic acid bacteria in bread dough enriched with zinc and selenium. Journal of Food Science and Engineering, 7, 497–504. 10.17265/2159-5828/2017.10.004

[fsn34050-bib-0058] Zörb, C. , Ludewig, U. , & Hawkesford, M. (2018). Perspective on wheat yield and quality with reduced nitrogen supply. Trends in Plant Science, 23, 1029–1037. 10.1016/j.tplants.2018.08.012 30249481 PMC6202697

