# Supplementary material for: Wholegrain triticale sourdough: Effects of triticale:Wheat flour ratio and hydration level on bread quality
Source: Food Sci Nutr. 2024 Mar 24;12(6):3910–9. doi: 10.1002/fsn3.4050 (PMC11167140; doi:10.1002/fsn3.4050)
Supplement: Supplementary file 1 — Table S1. [file FSN3-12-3910-s003.docx]

| Sample | *a** | *b** | *L** | BI | Chroma |
| --- | --- | --- | --- | --- | --- |
| WD | 2.21±0.01^d^ | 14.70±1.01^d^ | 73.20±2.31^a^ | 43.54±1.54^b^ | 17.21±1.10^b^ |
| GD | 8.23±0.04^b^ | 16.90±1.21^c^ | 58.61±3.10^c^ | 45.40±1.09^a^ | 17.91±1.32^b^ |
| HD | 9.08±0.02^a^ | 18.12±1.13^b^ | 55.16±2.34^d^ | 44.17±2.01^b^ | 18.61±1.09^a^ |
| SD | 5.98±0.01^c^ | 19.02±1.02^a^ | 60.62±3.21^b^ | 31.24±1.76^c^ | 18.83±1.21^a^ |
| *p*-value | 0.0001 | 0.0001 | 0.0001 | 0.0001 | 0.0001 |
| WD | 4.20±0.02^c^ | 16.31±1.02^b^ | 58.63±3.21^a^ | 61.31±2.22^a^ | 20.25±1.03^a^ |
| GC | 6.90±0.03^a^ | 18.12±1.10^a^ | 49.17±2.19^c^ | 57.30±2.37^b^ | 19.74±1.11^b^ |
| HC | 6.23±0.02^b^ | 18.25±1.21^a^ | 47.12±3.01^c^ | 56.22±3.02^b^ | 20.76±1.02^a^ |
| SC | 6.30±0.03^b^ | 18.96±1.12^a^ | 51.09±2.02^b^ | 40.51±1.98^c^ | 18.91±1.43^c^ |
| *p*-value | 0.0001 | 0.0001 | 0.0001 | 0.0001 | 0.0001 |
| WD | 13.79±1.01^a^ | 20.25±1.02^a^ | 41.53±3.11^c^ | 61.10±2.62^a^ | 24.50±1.67^a^ |
| GCT | 9.13±0.87^c^ | 9.10±.0.71^d^ | 48.66±3.21^b^ | 40.72±1.99^c^ | 14.26±1.03^b^ |
| HCT | 7.14±0.55^d^ | 11.29±0.98^c^ | 40.91±2.76^c^ | 35.11±2.03^d^ | 12.12±0.99^c^ |
| SCT | 11.23±0.92^b^ | 14.42±1.02^b^ | 51.85±2.47^a^ | 50.30±2.76^b^ | 8.37±0.76^d^ |
| *p*-value | 0.0001 | 0.0001 | 0.0001 | 0.0001 | 0.0001 |

Table S1. Colour parameters for sourdough bread dough, crumb and crust

of 100% wholegrain triticale and 100% wholemeal wheat flour.

*Means denoted by different letters in the columns indicate significant difference between samples (p<0.05) (Tukey’s test). GD, Goanna dough; GC, Goanna crumb; GCT, Goanna crust; HD, Hawkeye dough; HC, Hawkeye crumb; HCT, Hawkeye Crust; SD, Scout dough; SC, Scout crumb; SCT, Scout crust; *L**, lightness; *a**, redness/blueness; *b**, yellowness/greenness; BI, Brownness Index; NS, Not significant
