# Supplementary material for: Wholegrain triticale sourdough: Effects of triticale:Wheat flour ratio and hydration level on bread quality
Source: Food Sci Nutr. 2024 Mar 24;12(6):3910–9. doi: 10.1002/fsn3.4050 (PMC11167140; doi:10.1002/fsn3.4050)
Supplement: Supplementary file 2 — Table S2. [file FSN3-12-3910-s001.docx]

| Sample | Colour Parameter | | | | |
| --- | --- | --- | --- | --- | --- |
|  | *L** | *a** | *b** | BI | Chroma |
| GD_T1_ | 57.82±0.59^c^ | 6.05±0.34^a^ | 14.70±0.77^b^ | 43.54±0.05^a^ | 15.89±0.02^b^ |
| GD_T2_ | 58.32±0.61^c^ | 5.61±0.22^ab^ | 14.34±0.40^b^ | 37.64±0.18^b^ | 15.39±0.06^c^ |
| GD_T3_ | 60.58±1.24^b^ | 5.43±0.46^b^ | 13.81±1.14^b^ | 36.53±0.02^c^ | 14.84±0.10^d^ |
| GD_T4_ | 62.20±0.52^a^ | 5.80±0.28^ab^ | 16.72±0.75^a^ | 32.01±0.01^d^ | 17.69±0.04^a^ |
| p-value | 0.0001 | 0.034 | 0.0001 | 0.0001 | 0.0001 |
| HD_T1_ | 54.76±0.80^c^ | 6.84±0.44^a^ | 15.79±0.44^ab^ | 42.67±0.15^a^ | 17.21±0.46^a^ |
| HD_T2_ | 58.34±0.83^b^ | 6.38±0.15^b^ | 15.25±0.67^b^ | 37.84±0.21^b^ | 16.52±0.15^b^ |
| HD_T3_ | 62.27±0.75^a^ | 5.99±0.26^bc^ | 15.50±0.43^ab^ | 37.14±0.23^b^ | 17.46±0.23^a^ |
| HD_T4_ | 61.59±0.85^a^ | 5.71±0.19^c^ | 16.41±0.84^a^ | 35.32±0.14^c^ | 16.51±0.27^b^ |
| *p*-value | 0.0001 | 0.0001 | 0.0263 | 0.0001 | 0.0001 |
| SD_T1_ | 68.22±0.38 | 3.94±0.11^a^ | 15.69±0.26^c^ | 32.74±0.18^a^ | 17.63±0.21^a^ |
| SD_T2_ | 68.29±0.90 | 3.50±0.22^b^ | 16.81±0.79^b^ | 31.49±0.25^b^ | 17.17±0.11^a^ |
| SD_T3_ | 68.18±0.88 | 3.53±0.26^b^ | 16.43±0.36^b^ | 30.86±0.22^c^ | 16.80±0.25^b^ |
| SD_T4_ | 68.47±1.19 | 3.28±0.16^c^ | 17.19±0.20^a^ | 29.04±0.22^d^ | 16.80±0.04^b^ |
| *p*-value | NS | 0.0001 | 0.0002 | 0.0001 | 0.0001 |
| GCT_T1_ | 35.36±0.80^a^ | 8.73±0.28^c^ | 7.80±0.87^b^ | 34.81±0.14^d^ | 12.16±0.23^b^ |
| GCT_T2_ | 35.46±0.67^a^ | 9.34±0.61^b^ | 6.97±0.55^c^ | 40.54±0.19^c^ | 11.18±0.11^c^ |
| GCT_T3_ | 35.77±0.61^a^ | 10.27±0.93^a^ | 8.57±0.53^a^ | 48.06±0.13^b^ | 13.37±0.15^a^ |
| GCT_T4_ | 34.41±0.41^b^ | 10.05±0.66^a^ | 8.45±0.64^a^ | 46.76±0.17^a^ | 13.12±0.29^a^ |
| *p*-value | 0.0001 | 0.0001 | 0.0001 | 0.0001 | 0.0001 |
| HCT_T1_ | 44.76±0.16^a^ | 6.84±0.09^c^ | 8.79±0.02^a^ | 28.34±0.15^d^ | 10.82±0.31^c^ |
| HCT_T2_ | 43.35±0.87^b^ | 8.80±0.65^b^ | 6.17±1.10^c^ | 29.62±0.19^c^ | 10.74±0.30^c^ |
| HCT_T3_ | 42.75±1.07^c^ | 9.38±0.49^a^ | 6.81±1.15^c^ | 32.80±0.23^b^ | 11.59±0.30^b^ |
| HCT_T4_ | 43.14±1.11^b^ | 9.64±0.57^a^ | 7.42±0.87^b^ | 34.59±0.26^a^ | 12.16±0.25^a^ |
| *p*-value | 0.0001 | 0.0001 | 0.0001 | 0.0001 | 0.0001 |
| SCT_T1_ | 47.05±0.81^b^ | 10.83±1.13 | 11.79±1.14^b^ | 45.13±0.27^c^ | 16.00±0.46^c^ |
| SCT_T2_ | 49.40±1.06^a^ | 11.27±0.45 | 12.32±1.08^a^ | 45.66±0.21^c^ | 18.21±0.30^a^ |
| SCT_T3_ | 46.46±1.19^c^ | 11.44±0.83 | 11.44±1.04^b^ | 47.72±0.30^b^ | 16.17±0.29^c^ |
| SCT_T4_ | 48.10±1.00^b^ | 11.38±0.45 | 12.81±0.67^a^ | 50.35±0.27^a^ | 17.13±0.16^b^ |
| *p*-value | 0.0001 | NS | 0.0001 | 0.0001 | 0.0001 |
| GC_T1_ | 47.37±0.07^b^ | 5.90±0.05^a^ | 16.52±0.04^a^ | 51.30±0.01^a^ | 17.54±0.15^a^ |
| GC_T2_ | 45.64±0.72^b^ | 5.88±0.16^a^ | 13.71±0.40^b^ | 44.61±0.01^b^ | 14.91±0.12^c^ |
| GC_T3_ | 60.47±0.86^a^ | 3.11±0.41^b^ | 15.96±0.62^a^ | 34.24±0.19^c^ | 16.37±0.15^b^ |
| GC_T4_ | 61.11±1.04^a^ | 2.78±0.20^b^ | 16.61±0.29^a^ | 34.26±0.15^c^ | 16.49±0.35^b^ |
| *p*-value | 0.0001 | 0.0001 | 0.0001 | 0.0001 | 0.0001 |
| HC_T1_ | 45.07±0.05^c^ | 5.83±0.04 | 16.15±0.02^a^ | 50.00±0.01^a^ | 19.06±0.05^a^ |
| HC_T2_ | 50.81±0.73^b^ | 6.13±0.19 | 13.44±0.42^c^ | 39.08±0.14^b^ | 14.76±0.33^c^ |
| HC_T3_ | 51.06±0.24^b^ | 5.96±0.25 | 13.23±0.25^c^ | 40.07±0.18^b^ | 14.50±0.20^c^ |
| HC_T4_ | 52.56±0.26^a^ | 5.84±0.08 | 14.64±0.19^b^ | 40.27±0.13^b^ | 15.75±0.21^b^ |
| *p*-value | 0.0001 | NS | 0.0001 | 0.0001 | NS |
| SC_T1_ | 60.83±0.70^b^ | 2.80±0.17^b^ | 16.66±0.29 | 36.09±0.22^a^ | 16.89±0.33^b^ |
| SC_T2_ | 60.89±0.50^b^ | 3.16±0.37^a^ | 16.02±0.58 | 34.12±0.21^b^ | 16.32±0.39^b^ |
| SC_T3_ | 61.39±0.67^a^ | 3.21±0.11^a^ | 16.70±0.65 | 34.05±0.12^b^ | 17.00±0.27^a^ |
| SC_T4_ | 61.80±0.85^a^ | 3.33±0.12^a^ | 16.99±0.32 | 34.99±0.44^b^ | 17.30±0.12^a^ |
| *p*-value | 0.0001 | 0.0001 | NS | 0.0001 | 0.0001 |

Table S2. Colour parameters for sourdough bread dough, crumb and crust with different ratios of wholegrain triticale or wheat flour to wholemeal wheat flour.

*****Means denoted by different letters in the columns indicate significant difference between samples (*p*<0.05) (Tukey’s test). GD, Goanna dough; GC, Goanna crumb; GCT, Goanna crust; HD, Hawkeye dough; HC, Hawkeye crumb; HCT Hawkeye Crust; SD, Scout dough; SC, Scout crumb; SCT, Scout crust; T_1_, 90% of wholegrain triticale or wheat flour and 10% wholemeal wheat flour; T_2_, 80% wholegrain triticale or wheat flour and 20% wholemeal wheat flour; T_3_, 70% wholegrain triticale or wheat flour and 30% wholemeal wheat flour; T_4_, 60% wholegrain triticale or wheat flour and 40% wholemeal wheat flour; *L**, lightness; *a**, redness/blueness; *b**, yellowness/greenness; BI, Brownness Index; NS, Not significant.
