# Supplementary material for: Wholegrain triticale sourdough: Effects of triticale:Wheat flour ratio and hydration level on bread quality
Source: Food Sci Nutr. 2024 Mar 24;12(6):3910–9. doi: 10.1002/fsn3.4050 (PMC11167140; doi:10.1002/fsn3.4050)
Supplement: Supplementary file 3 — Table S3. [file FSN3-12-3910-s002.docx]

Table S3. Colour parameters for sourdough bread dough crumb and crust with varying dough water content.

| Sample | Colour Parameter | | | | | | | | | |
| --- | --- | --- | --- | --- | --- | --- | --- | --- | --- | --- |
|  | *L** | | *a** | | *b** | | BI | | | Chroma |
| GD_W1_ | 59.18±1.25^b^ | | 6.59±0.21^ab^ | | 15.23±0.45^a^ | | 37.47±0.34^a^ | | | 17.60±0.28^a^ |
| GD_W2_ | 58.98±0.66^b^ | | 6.53±0.29^ab^ | | 15.16±0.40^ab^ | | 37.37±0.27^a^ | | | 16.50±0.33^a^ |
| GD_W3_ | 58.78±0.71^b^ | | 6.68±0.41^a^ | | 15.08±0.70^ab^ | | 37.55±0.28^a^ | | | 16.50±0.18^a^ |
| GD_W4_ | 61.09±0.11^a^ | | 6.16±0.06^b^ | | 14.44±0.23^b^ | | 33.95±0.35^b^ | | | 15.71±0.25^b^ |
| p-value | 0.0001 | | 0.0001 | | 0.0001 | | 0.0001 | | | 0.0001 |
| HD_W1_ | 55.24±0.80^b^ | | 7.51±0.26^a^ | | 15.90±0.18^a^ | | 43.41±0.17^a^ | | | 17.58±0.01^a^ |
| HD_W2_ | 57.85±0.80^a^ | | 7.23±0.11^b^ | | 15.75±0.24^a^ | | 40.48±0.33^b^ | | | 17.34±0.11^a^ |
| HD_W3_ | 57.26±0.57^a^ | | 6.71±0.13^c^ | | 14.99±0.40^b^ | | 38.48±0.31^c^ | | | 16.42±0.39^b^ |
| HD_W4_ | 57.04±0.55^a^ | | 6.70±0.05^c^ | | 14.44±0.31^c^ | | 37.37±0.30^d^ | | | 15.92±0.39^c^ |
| *p*-value | 0.0001 | | 0.0001 | | 0.0001 | | 0.0001 | | | 0.0001 |
| SD_W1_ | 67.46±1.12 | | 3.83±0.39 | | 17.44±0.58^ab^ | | 33.67±0.38^a^ | | | 17.86±0.39^b^ |
| SD_W2_ | 67.54±0.72 | | 3.53±0.13 | | 17.02±0.26^ab^ | | 32.34±0.25^b^ | | | 17.39±0.31^b^ |
| SD_W3_ | 67.97±1.36 | | 3.79±0.37 | | 16.85±0.47^b^ | | 32.07±0.36^b^ | | | 17.27±0.05^b^ |
| SD_W4_ | 67.84±0.40 | | 3.81±0.18 | | 17.63±0.31^a^ | | 32.54±0.38^b^ | | | 18.04±0.38^a^ |
| *p*-value | NS | | NS | | 0.0001 | | 0.0001 | | | 0.0001 |
| GCT_W1_ | 42.94±0.41^a^ | | 8.99±0.26^b^ | | 5.63±0.37^b^ | | 33.71±0.33^a^ | | | 10.61±0.20^b^ |
| GCT_W2_ | 42.89±0.24^a^ | | 8.92±0.26^b^ | | 7.02±0.32^a^ | | 29.42±0.31^b^ | | | 11.93±0.39^a^ |
| GCT_W3_ | 42.26±0.41^b^ | | 9.04±0.19^b^ | | 5.51±0.37^b^ | | 29.12±0.32^b^ | | | 10.49±0.13^b^ |
| GCT_W4_ | 41.85±0.21 ^b^ | | 9.64±0.21^a^ | | 5.45±0.48^b^ | | 28.80±0.35^c^ | | | 10.56±0.67^b^ |
| *p*-value | | 0.0001 | | 0.0001 | | 0.0001 | | 0.0001 | 0.0001 | |
| HCT_W1_ | | 44.01±0.39^a^ | | 9.82±0.40 | | 6.98±0.50^b^ | | 43.41±0.32^a^ | 12.69±0.27^b^ | |
| HCT_W2_ | | 43.45±0.34^a^ | | 9.76±0.18 | | 8.04±0.54^a^ | | 40.48±0.30^c^ | 12.01±0.31^b^ | |
| HCT_W3_ | | 41.17±0.23^b^ | | 9.83±0.18 | | 8.02±0.31^a^ | | 41.22±0.33^b^ | 12.73±0.21^b^ | |
| HCT_W4_ | | 41.71±0.59^b^ | | 10.25±0.41 | | 8.86±0.98^a^ | | 38.68±0.68^d^ | 13.55±0.21^a^ | |
| *p*-value | | 0.0001 | | NS | | 0.0007 | | 0.0001 | 0.0001 | |
| SCT_W1_ | | 45.43±0.38 | | 0.52±0.44^c^ | | 9.42±0.96^b^ | | 42.81±0.35^a^ | 14.12±0.64^b^ | |
| SCT_W2_ | | 45.60±0.38 | | 10.10±0.57^b^ | | 9.58±0.40^b^ | | 40.11±0.35^b^ | 13.93±0.74^b^ | |
| SCT_W3_ | | 46.33±0.64 | | 11.11±0.24^a^ | | 9.64±0.86^b^ | | 40.68±0.15^b^ | 16.07±0.36^a^ | |
| SCT_W4_ | | 45.88±0.75 | | 10.83±0.41^a^ | | 11.60±0.43^a^ | | 39.25±0.33^c^ | 14.501±0.29^b^ | |
| *p*-value | | NS | | 0.0001 | | 0.0001 | | 0.0001 | 0.0001 | |
| GC_W1_ | | 48.45±0.88 ^b^ | | 5.94±0.04 | | 10.54±0.47^b^ | | 35.09±0.74^a^ | 12.10±0.40^a^ | |
| GC_W2_ | | 48.38±0.42 ^b^ | | 6.13±0.19 | | 11.06±0.13^b^ | | 35.40±0.14^a^ | 12.65±0.21^a^ | |
| GC_W3_ | | 48.41±0.60 ^b^ | | 6.25±0.21 | | 11.10±0.93^b^ | | 34.05±0.31^b^ | 12.74±0.41^a^ | |
| GC_W4_ | | 50.12±0.35^a^ | | 6.05±0.13 | | 11.51±0.06^a^ | | 33.11±0.16^c^ | 13.00±0.40^a^ | |
| *p*-value | | 0.0001 | | NS | | 0.0001 | | 0.0001 | 0.0001 | |
| HC_W1_ | | 48.22±0.22^b^ | | 6.69±0.10^a^ | | 13.95±0.11^a^ | | 40.79±0.33^a^ | 15.47±0.192^a^ | |
| HC_W2_ | | 48.99±0.56^b^ | | 6.22±0.07^b^ | | 12.18±0.11^b^ | | 38.17±0.14^b^ | 13.68±0.28^c^ | |
| HC_W3_ | | 48.83±0.47^b^ | | 6.05±0.16^b^ | | 12.87±0.38^b^ | | 37.47±0.16^c^ | 14.22±0.09^b^ | |
| HC_W4_ | | 50.02±0.24^a^ | | 6.15±0.08^b^ | | 12.32±0.40^b^ | | 37.88±0.38^c^ | 13.77±0.29^c^ | |
| *p*-value | | 0.0001 | | 0.0001 | | 0.0001 | | 0.0001 | 0.0001 | |
| SC_W1_ | | 57.94±0.95^b^ | | 4.18±0.09^a^ | | 19.54±0.55^a^ | | 45.64±0.50^a^ | 19.98±0.53^a^ | |
| SC_W2_ | | 59.07±0.17^a^ | | 4.07±0.15^a^ | | 18.86±0.05^ab^ | | 42.34±0.73^b^ | 19.22±0.16^a^ | |
| SC_W3_ | | 59.15±0.23^a^ | | 3.72±0.02^b^ | | 19.25±0.19^a^ | | 43.66±0.33^b^ | 19.67±0.51^ab^ | |
| SC_W4_ | | 59.53±0.17^a^ | | 3.54±0.09^b^ | | 18.17±0.38^b^ | | 40.10±0.40^c^ | 18.52±0.20^b^ | |
| *p*-value | | 0.0001 | | 0.0001 | | 0.0001 | | 0.0001 | 0.0001 | |

*****Means denoted by different letters in the columns indicate significant difference between samples (*p*<0.05) (Tukey’s test). GD, Goanna dough; GC, Goanna crumb; GCT, Goanna crust; HD, Hawkeye dough; HC, Hawkeye crumb; HCT, Hawkeye crust; SD, Scout dough; SC, Scout crumb; SCT, Scout crust; W_1_, 70% water; W_2_, 80% water; W_3,_ 90% water; W_4_, 100% water; *L**, lightness; *a**, redness/blueness; *b**, yellowness/greenness; BI, Brownness Index; NS, not significant.
